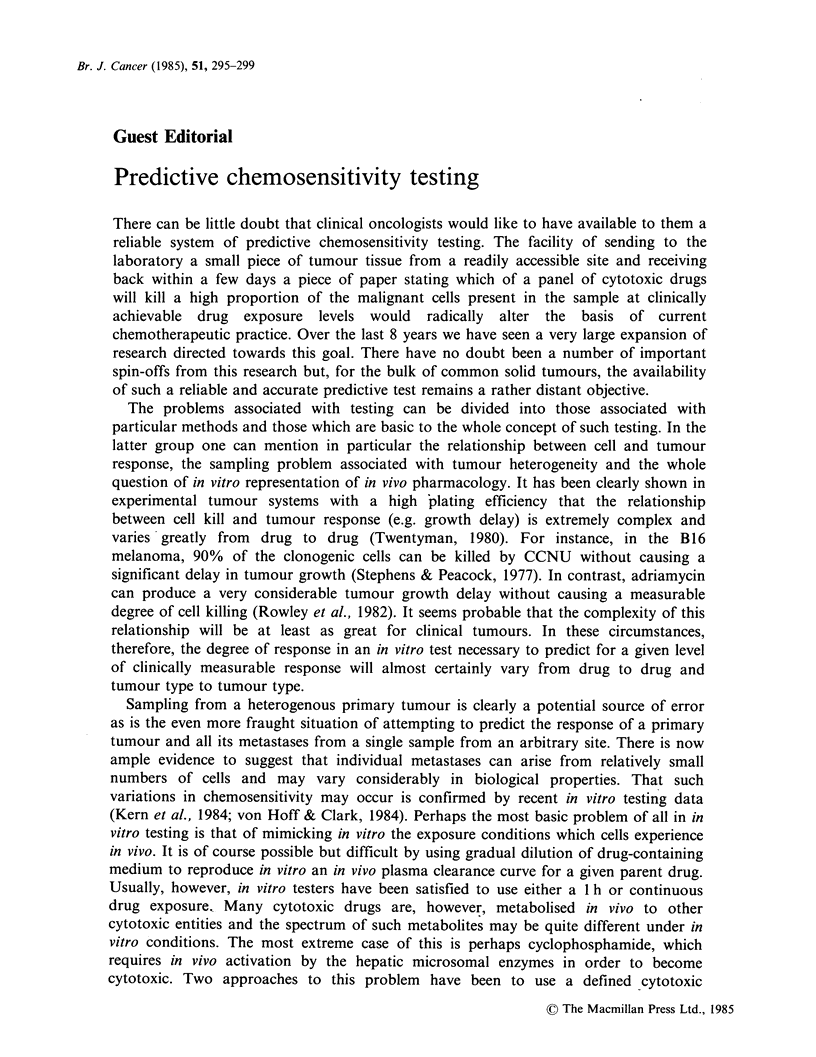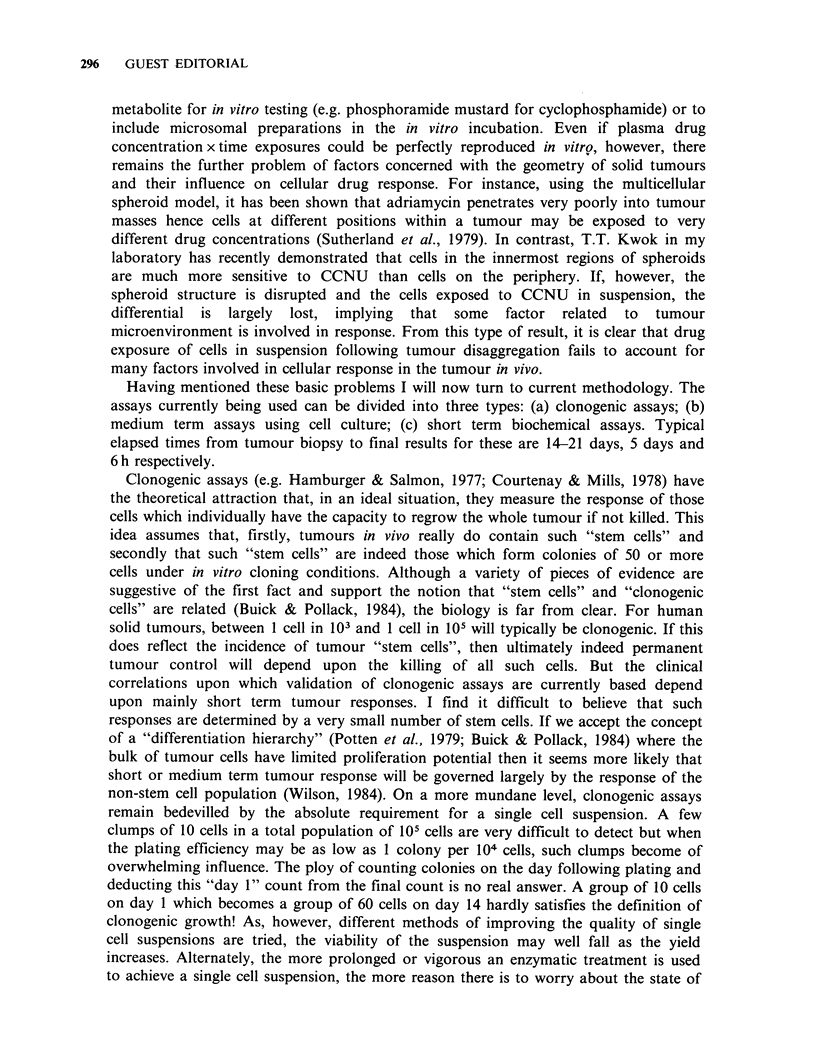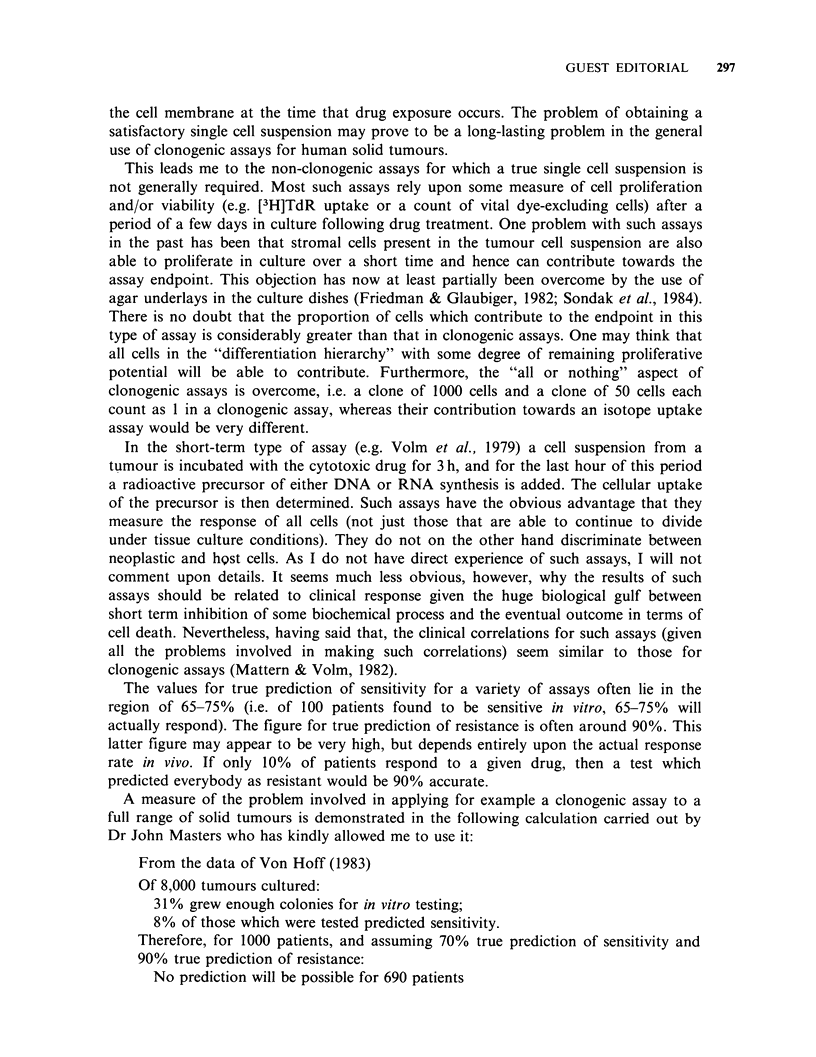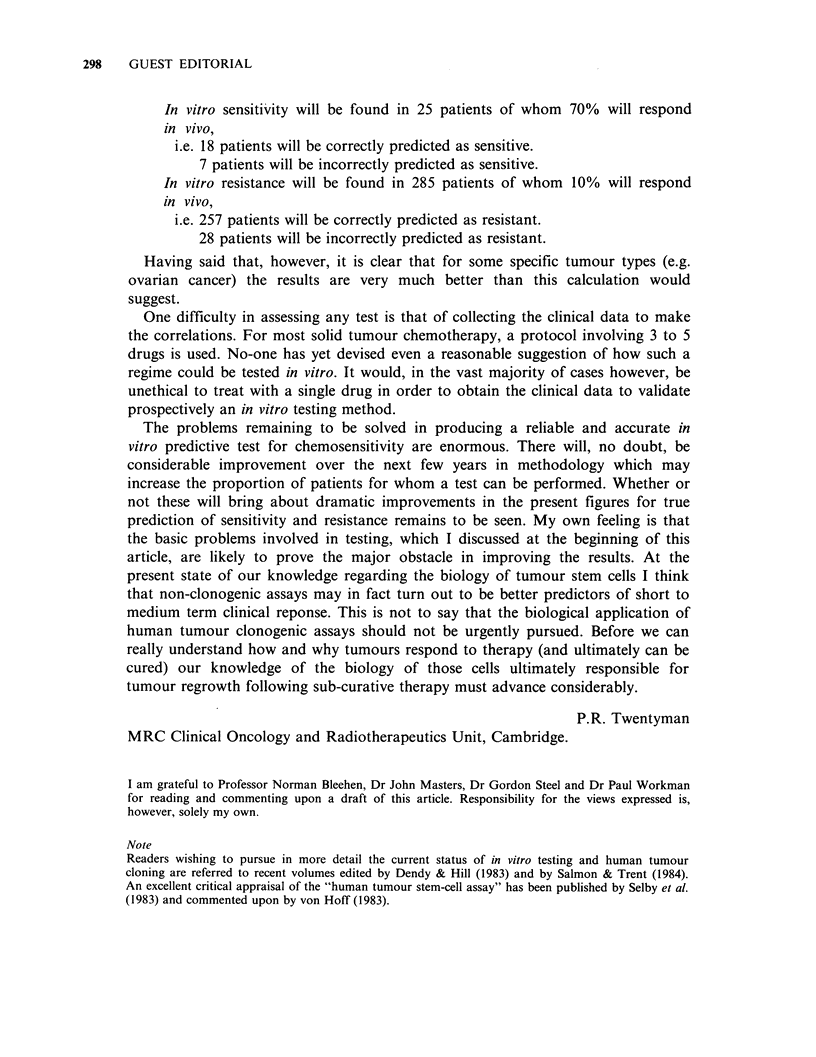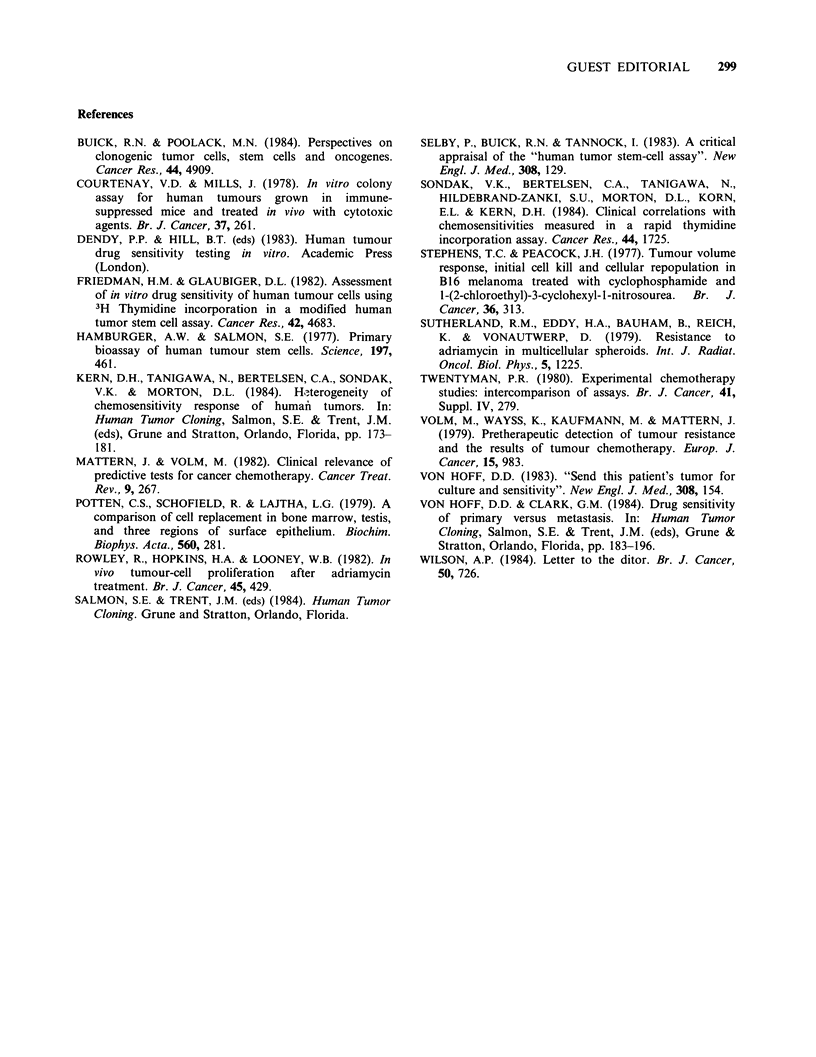# Predictive chemosensitivity testing.

**DOI:** 10.1038/bjc.1985.42

**Published:** 1985-03

**Authors:** P. R. Twentyman


Br. J. Cancer (1985), 51, 295–299

### Guest Editorial

### Predictive chemosensitivity testing

There can be little doubt that clinical oncologists would like to have available to them a reliable system of predictive chemosensitivity testing. The facility of sending to the laboratory a small piece of tumour tissue from a readily accessible site and receiving back within a few days a piece of paper stating which of a panel of cytotoxic drugs will kill a high proportion of the malignant cells present in the sample at clinically achievable drug exposure levels would radically alter the basis of current chemotherapeutic practice. Over the last 8 years we have seen a very large expansion of research directed towards this goal. There have no doubt been a number of important spin-offs from this research but, for the bulk of common solid tumours, the availability of such a reliable and accurate predictive test remains a rather distant objective.

The problems associated with testing can be divided into those associated with particular methods and those which are basic to the whole concept of such testing. In the latter group one can mention in particular the relationship between cell and tumour response, the sampling problem associated with tumour heterogeneity and the whole question of *in vitro* representation of *in vivo* pharmacology. It has been clearly shown in experimental tumour systems with a high plating efficiency that the relationship between cell kill and tumour response (e.g. growth delay) is extremely complex and varies greatly from drug to drug (Twentyman, 1980). For instance, in the B16 melanoma, 90% of the clonogenic cells can be killed by CCNU without causing a significant delay in tumour growth (Stephens & Peacock, 1977). In contrast, adriamycin can produce a very considerable tumour growth delay without causing a measurable degree of cell killing (Rowley *et al.*, 1982). It seems probable that the complexity of this relationship will be at least as great for clinical tumours. In these circumstances, therefore, the degree of response in an *in vitro* test necessary to predict for a given level of clinically measurable response will almost certainly vary from drug to drug and tumour type to tumour type.

Sampling from a heterogenous primary tumour is clearly a potential source of error as is the even more fraught situation of attempting to predict the response of a primary tumour and all its metastases from a single sample from an arbitrary site. There is now ample evidence to suggest that individual metastases can arise from relatively small numbers of cells and may vary considerably in biological properties. That such variations in chemosensitivity may occur is confirmed by recent *in vitro* testing data (Kern *et al.*, 1984; von Hoff & Clark, 1984). Perhaps the most basic problem of all in *in vitro* testing is that of mimicking *in vitro* the exposure conditions which cells experience *in vivo*. It is of course possible but difficult by using gradual dilution of drug-containing medium to reproduce *in vitro* an *in vivo* plasma clearance curve for a given parent drug. Usually, however, *in vitro* testers have been satisfied to use either a 1 h or continuous drug exposure. Many cytotoxic drugs are, however, metabolised *in vivo* to other cytotoxic entities and the spectrum of such metabolites may be quite different under *in vitro* conditions. The most extreme case of this is perhaps cyclophosphamide, which requires *in vivo* activation by the hepatic microsomal enzymes in order to become cytotoxic. Two approaches to this problem have been to use a defined cytotoxic

© The Macmillan Press Ltd., 1985

metabolite for *in vitro* testing (e.g. phosphoramide mustard for cyclophosphamide) or to include microsomal preparations in the *in vitro* incubation. Even if plasma drug concentration × time exposures could be perfectly reproduced *in vitro*, however, there remains the further problem of factors concerned with the geometry of solid tumours and their influence on cellular drug response. For instance, using the multicellular spheroid model, it has been shown that adriamycin penetrates very poorly into tumour masses hence cells at different positions within a tumour may be exposed to very different drug concentrations (Sutherland *et al.*, 1979). In contrast, T.T. Kwok in my laboratory has recently demonstrated that cells in the innermost regions of spheroids are much more sensitive to CCNU than cells on the periphery. If, however, the spheroid structure is disrupted and the cells exposed to CCNU in suspension, the differential is largely lost, implying that some factor related to tumour microenvironment is involved in response. From this type of result, it is clear that drug exposure of cells in suspension following tumour disaggregation fails to account for many factors involved in cellular response in the tumour *in vivo*.

Having mentioned these basic problems I will now turn to current methodology. The assays currently being used can be divided into three types: (a) clonogenic assays; (b) medium term assays using cell culture; (c) short term biochemical assays. Typical elapsed times from tumour biopsy to final results for these are 14–21 days, 5 days and 6 h respectively.

Clonogenic assays (e.g. Hamburger & Salmon, 1977; Courtenay & Mills, 1978) have the theoretical attraction that, in an ideal situation, they measure the response of those cells which individually have the capacity to regrow the whole tumour if not killed. This idea assumes that, firstly, tumours *in vivo* really do contain such "stem cells" and secondly that such "stem cells" are indeed those which form colonies of 50 or more cells under *in vitro* cloning conditions. Although a variety of pieces of evidence are suggestive of the first fact and support the notion that "stem cells" and "clonogenic cells" are related (Buick & Pollack, 1984), the biology is far from clear. For human solid tumours, between 1 cell in $10^3$ and 1 cell in $10^5$ will typically be clonogenic. If this does reflect the incidence of tumour "stem cells", then ultimately indeed permanent tumour control will depend upon the killing of all such cells. But the clinical correlations upon which validation of clonogenic assays are currently based depend upon mainly short term tumour responses. I find it difficult to believe that such responses are determined by a very small number of stem cells. If we accept the concept of a "differentiation hierarchy" (Potten *et al.*, 1979; Buick & Pollack, 1984) where the bulk of tumour cells have limited proliferation potential then it seems more likely that short or medium term tumour response will be governed largely by the response of the non-stem cell population (Wilson, 1984). On a more mundane level, clonogenic assays remain bedevilled by the absolute requirement for a single cell suspension. A few clumps of 10 cells in a total population of $10^5$ cells are very difficult to detect but when the plating efficiency may be as low as 1 colony per $10^4$ cells, such clumps become of overwhelming influence. The ploy of counting colonies on the day following plating and deducting this "day 1" count from the final count is no real answer. A group of 10 cells on day 1 which becomes a group of 60 cells on day 14 hardly satisfies the definition of clonogenic growth! As, however, different methods of improving the quality of single cell suspensions are tried, the viability of the suspension may well fall as the yield increases. Alternately, the more prolonged or vigorous an enzymatic treatment is used to achieve a single cell suspension, the more reason there is to worry about the state of the cell membrane at the time that drug exposure occurs. The problem of obtaining a satisfactory single cell suspension may prove to be a long-lasting problem in the general use of clonogenic assays for human solid tumours.

This leads me to the non-clonogenic assays for which a true single cell suspension is not generally required. Most such assays rely upon some measure of cell proliferation and/or viability (e.g. [$^3$H]TdR uptake or a count of vital dye-excluding cells) after a period of a few days in culture following drug treatment. One problem with such assays in the past has been that stromal cells present in the tumour cell suspension are also able to proliferate in culture over a short time and hence can contribute towards the assay endpoint. This objection has now at least partially been overcome by the use of agar underlays in the culture dishes (Friedman & Glaubiger, 1982; Sondak *et al.*, 1984). There is no doubt that the proportion of cells which contribute to the endpoint in this type of assay is considerably greater than that in clonogenic assays. One may think that all cells in the "differentiation hierarchy" with some degree of remaining proliferative potential will be able to contribute. Furthermore, the "all or nothing" aspect of clonogenic assays is overcome, i.e. a clone of 1000 cells and a clone of 50 cells each count as 1 in a clonogenic assay, whereas their contribution towards an isotope uptake assay would be very different.

In the short-term type of assay (e.g. Volm *et al.,* 1979) a cell suspension from a tumour is incubated with the cytotoxic drug for 3 h, and for the last hour of this period a radioactive precursor of either DNA or RNA synthesis is added. The cellular uptake of the precursor is then determined. Such assays have the obvious advantage that they measure the response of all cells (not just those that are able to continue to divide under tissue culture conditions). They do not on the other hand discriminate between neoplastic and host cells. As I do not have direct experience of such assays, I will not comment upon details. It seems much less obvious, however, why the results of such assays should be related to clinical response given the huge biological gulf between short term inhibition of some biochemical process and the eventual outcome in terms of cell death. Nevertheless, having said that, the clinical correlations for such assays (given all the problems involved in making such correlations) seem similar to those for clonogenic assays (Mattern & Volm, 1982).

The values for true prediction of sensitivity for a variety of assays often lie in the region of 65–75% (i.e. of 100 patients found to be sensitive *in vitro*, 65–75% will actually respond). The figure for true prediction of resistance is often around 90%. This latter figure may appear to be very high, but depends entirely upon the actual response rate *in vivo*. If only 10% of patients respond to a given drug, then a test which predicted everybody as resistant would be 90% accurate.

A measure of the problem involved in applying for example a clonogenic assay to a full range of solid tumours is demonstrated in the following calculation carried out by Dr John Masters who has kindly allowed me to use it:

From the data of Von Hoff (1983)

Of 8,000 tumours cultured:

31% grew enough colonies for *in vitro* testing;

8% of those which were tested predicted sensitivity.

Therefore, for 1000 patients, and assuming 70% true prediction of sensitivity and 90% true prediction of resistance:

No prediction will be possible for 690 patients

*In vitro* sensitivity will be found in 25 patients of whom 70% will respond *in vivo*,
i.e. 18 patients will be correctly predicted as sensitive.
7 patients will be incorrectly predicted as sensitive.
*In vitro* resistance will be found in 285 patients of whom 10% will respond *in vivo*,
i.e. 257 patients will be correctly predicted as resistant.
28 patients will be incorrectly predicted as resistant.

Having said that, however, it is clear that for some specific tumour types (e.g. ovarian cancer) the results are very much better than this calculation would suggest.

One difficulty in assessing any test is that of collecting the clinical data to make the correlations. For most solid tumour chemotherapy, a protocol involving 3 to 5 drugs is used. No-one has yet devised even a reasonable suggestion of how such a regime could be tested *in vitro*. It would, in the vast majority of cases however, be unethical to treat with a single drug in order to obtain the clinical data to validate prospectively an *in vitro* testing method.

The problems remaining to be solved in producing a reliable and accurate *in vitro* predictive test for chemosensitivity are enormous. There will, no doubt, be considerable improvement over the next few years in methodology which may increase the proportion of patients for whom a test can be performed. Whether or not these will bring about dramatic improvements in the present figures for true prediction of sensitivity and resistance remains to be seen. My own feeling is that the basic problems involved in testing, which I discussed at the beginning of this article, are likely to prove the major obstacle in improving the results. At the present state of our knowledge regarding the biology of tumour stem cells I think that non-clonogenic assays may in fact turn out to be better predictors of short to medium term clinical reponse. This is not to say that the biological application of human tumour clonogenic assays should not be urgently pursued. Before we can really understand how and why tumours respond to therapy (and ultimately can be cured) our knowledge of the biology of those cells ultimately responsible for tumour regrowth following sub-curative therapy must advance considerably.

P.R. Twentyman
MRC Clinical Oncology and Radiotherapeutics Unit, Cambridge.

I am grateful to Professor Norman Bleehen, Dr John Masters, Dr Gordon Steel and Dr Paul Workman for reading and commenting upon a draft of this article. Responsibility for the views expressed is, however, solely my own.

*Note*
Readers wishing to pursue in more detail the current status of *in vitro* testing and human tumour cloning are referred to recent volumes edited by Dendy & Hill (1983) and by Salmon & Trent (1984). An excellent critical appraisal of the "human tumour stem-cell assay" has been published by Selby *et al.* (1983) and commented upon by von Hoff (1983).

**References**

BUICK, R.N. & POOLACK, M.N. (1984). Perspectives on clonogenic tumor cells, stem cells and oncogenes. *Cancer Res.*, **44**, 4909.

COURTENAY, V.D. & MILLS, J. (1978). *In vitro* colony assay for human tumours grown in immune-suppressed mice and treated *in vivo* with cytotoxic agents. *Br. J. Cancer*, **37**, 261.

DENDY, P.P. & HILL, B.T. (eds) (1983). Human tumour drug sensitivity testing *in vitro*. Academic Press (London).

FRIEDMAN, H.M. & GLAUBIGER, D.L. (1982). Assessment of *in vitro* drug sensitivity of human tumour cells using $^{3}H$ Thymidine incorporation in a modified human tumor stem cell assay. *Cancer Res.*, **42**, 4683.

HAMBURGER, A.W. & SALMON, S.E. (1977). Primary bioassay of human tumour stem cells. *Science*, **197**, 461.

KERN, D.H., TANIGAWA, N., BERTELSEN, C.A., SONDAK, V.K. & MORTON, D.L. (1984). Heterogeneity of chemosensitivity response of human tumors. In: *Human Tumor Cloning*, Salmon, S.E. & Trent, J.M. (eds), Grune and Stratton, Orlando, Florida, pp. 173–181.

MATTERN, J. & VOLM, M. (1982). Clinical relevance of predictive tests for cancer chemotherapy. *Cancer Treat. Rev.*, **9**, 267.

POTTEN, C.S., SCHOFIELD, R. & LAJTHA, L.G. (1979). A comparison of cell replacement in bone marrow, testis, and three regions of surface epithelium. *Biochim. Biophys. Acta.*, **560**, 281.

ROWLEY, R., HOPKINS, H.A. & LOONEY, W.B. (1982). *In vivo* tumour-cell proliferation after adriamycin treatment. *Br. J. Cancer*, **45**, 429.

SALMON, S.E. & TRENT, J.M. (eds) (1984). *Human Tumor Cloning*. Grune and Stratton, Orlando, Florida.

SELBY, P., BUICK, R.N. & TANNOCK, I. (1983). A critical appraisal of the "human tumor stem-cell assay". *New Engl. J. Med.*, **308**, 129.

SONDAK, V.K., BERTELSEN, C.A., TANIGAWA, N., HILDEBRAND-ZANKI, S.U., MORTON, D.L., KORN, E.L. & KERN, D.H. (1984). Clinical correlations with chemosensitivities measured in a rapid thymidine incorporation assay. *Cancer Res.*, **44**, 1725.

STEPHENS, T.C. & PEACOCK, J.H. (1977). Tumour volume response, initial cell kill and cellular repopulation in B16 melanoma treated with cyclophosphamide and 1-(2-chloroethyl)-3-cyclohexyl-1-nitrosourea. *Br. J. Cancer*, **36**, 313.

SUTHERLAND, R.M., EDDY, H.A., BAUHAM, B., REICH, K. & VONAUTWERP, D. (1979). Resistance to adriamycin in multicellular spheroids. *Int. J. Radiat. Oncol. Biol. Phys.*, **5**, 1225.

TWENTYMAN, P.R. (1980). Experimental chemotherapy studies: intercomparison of assays. *Br. J. Cancer*, **41**, Suppl. IV, 279.

VOLM, M., WAYSS, K., KAUFMANN, M. & MATTERN, J. (1979). Pretherapeutic detection of tumour resistance and the results of tumour chemotherapy. *Europ. J. Cancer*, **15**, 983.

VON HOFF, D.D. (1983). "Send this patient's tumor for culture and sensitivity". *New Engl. J. Med.*, **308**, 154.

VON HOFF, D.D. & CLARK, G.M. (1984). Drug sensitivity of primary versus metastasis. In: *Human Tumor Cloning*, Salmon, S.E. & Trent, J.M. (eds), Grune & Stratton, Orlando, Florida, pp. 183–196.

WILSON, A.P. (1984). Letter to the ditor. *Br. J. Cancer*, **50**, 726.